# Effect of grelin on TRX expression in chronic heart failure tissue: A protocol of systematic review and meta-analysis

**DOI:** 10.1097/MD.0000000000020294

**Published:** 2020-05-22

**Authors:** Zhe Chen, Yao Feng, Ru-bing Zhang, Xin Li, Jian-bo Xu

**Affiliations:** a Department of Critical Medicine; b Department of Acupuncture and Moxibustion; c Third Ward of Cardiology Department, First Affiliated Hospital of Jiamusi University; d Department of General Medicine, Jiamusi Central Hospital, Jiamusi, China.

**Keywords:** chronic heart failure, effect, grelin, TRX expression

## Abstract

**Background:**

The aim of this study is to explore the effect of grelin on TRX expression (TRXE) in chronic heart failure tissue (CHFT).

**Methods:**

We will search electronic databases from inception to the March 1, 2020 in MEDLINE, EMBASE, Cochrane Library, CINAHL, PEDro, the Allied and Complementary Medicine Database, Chinese Biomedical Literature Database, and China National Knowledge Infrastructure. We will not apply any limitations to the language and publication status. Any randomized controlled trials (RCTs) that studied the effect of grelin on TRXE in CHFT will be included. Study quality will be checked by Cochrane risk of bias and evidence quality will be appraised by Grading of Recommendations Assessment Development and Evaluation. All extracted data will be analyzed by RevMan 5.3 Software.

**Results:**

This study will summarize the present RCTs to assess the effect of grelin on TRXE in CHFT.

**Conclusion:**

The results of this study will provide conclusive evidence of the effect of grelin on TRXE in CHFT.

**Systematic review registration:**

INPLASY202040078.

## 1 Introduction

Chronic heart failure (CHF) is a progressive cardiovascular disease,^[1–3]^ which is characterized by abnormal cardiac structure or function.^[4–6]^ It is the leading cause of global disability and mortality.^[7–9]^ It is estimated that it affects 1% and 2% of adults, and more than 10% of patients over 70 years old.^[10]^ Thus, it is very essential to diagnose CHF at early stage. Previous studies have found that biomarkers, such as TRX expression (TRXE) in the heart tissue can help to diagnose CHF.^[11–22]^ However, no systematic review has been published to asses the effect of grelin on TRXE in chronic heart failure tissue (CHFT). To address the lack of conclusive evidence of the effect of grelin on TRXE in CHFT, this study aims to evaluate the randomized controlled trials (RCTs) exclusively assessed TRXE of CHFT managed by grelin.

## 2 Methods and analysis

### 2.1 Study registration

We have registered this study through INPLASY202040078, and we have reported it following the guideline of Preferred Reporting Items for Systematic Reviews and Meta-Analysis Protocol statement.^[23]^

### 2.2 Dissemination and ethics

We will publish this study through a peer-reviewed journal or a related conference. This study will not require ethic approval, since no privacy data will be obtained.

### 2.3 Eligibility criteria

#### 2.3.1 Participants/subjects

In this study, we will select CHFT as our research target.

#### 2.3.2 Interventions/exposure

Any types of grelin will be used for the treatment in the interventional group.

Any managements (such as no treatment, inhibitor) will be utilized as a comparator in the control group. We will exclude studies that used any forms of grelin as their controls.

#### 2.3.3 Study types

This study will include RCTs of grelin on TRXE in CHFT, regardless language and publication status limitations.

#### 2.3.4 Outcome measurements

Primary outcome are protein and gene expressions of TRXE. Protein expression of TRXE is measured by any related test, such as immunohistochemistry. Gene expression of TRXE is detected by reverse transcription polymerase chain reaction test or other related tests.

Secondary outcomes include left ventricular end-diastolic diameter, left ventricular end-systolic diameter, end-diastolic left ventricular posterior wall thickness, left ventricular ejection fraction, left ventricular systolic blood pressure, left ventricular end diastolic pressure, maximum left ventricular pressure increase rate, and maximum left ventricular pressure decrease rate.

### 2.4 Literature search

The following databases will be utilized to retrieve relevant studies from inception to the March 1, 2020 in MEDLINE, EMBASE, Cochrane Library, CINAHL, PEDro, the Allied and Complementary Medicine Database, Chinese Biomedical Literature Database, and China National Knowledge Infrastructure. We will not place any restrictions to the language and publication status. We have built a search strategy sample of Cochrane Library (Table 1). The equivalent search strategies will be modified for other electronic databases.

**Table 1:**
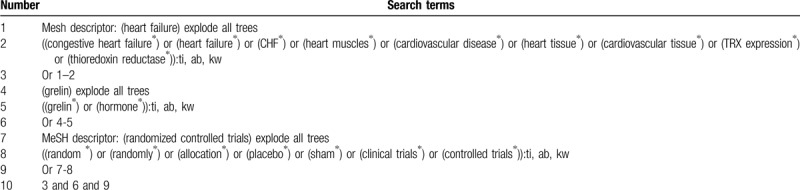
Search strategy of Cochrane Library.

We will also search secondary literature sources, such as Google scholar, website of clinical trial registries, and reference lists of relevant reviews.

### 2.5 Literature selection

NoteExpress 3.2.0 will be utilized to manage all searched records, and duplicated studies will be removed. Two researchers will independently scrutinize titles/ abstracts of potential studies to exclude all irrelevant ones. After that, full-text of remaining articles will be read cautiously against all inclusion criteria, and eligible trials will be included finally. If any conflicts are identified between two researchers, we will invite a third researcher to help solve them through discussion. The results of literature selection will be presented in a flow diagram.

### 2.6 Data extraction and management

Two researchers will separately perform data collection using pre-constructed data extraction sheet. It includes publication information (title, first author, year of publication, et al), targeted subject, sample size, study methods (randomization, blind, et al), details of interventions and controls (types of managements, dosage, et al), outcomes, results, conclusions, conflict of interest, and other related information. Any disagreements will be settled by a third researcher via discussion. If we identify any unclear or missing information, we will contact primary authors to request it.

### 2.7 Risk of bias assessment

Two researchers will separately assess study quality of all included studies using Cochrane risk of bias tool. It covers seven items, and each item is rated as low, unclear, or high risk of bias. A third researcher will be invited to tackle any differences through discussion.

### 2.8 Statistical analysis

#### 2.8.1 Data synthesis

The statistical analysis will be undertaken by RevMan 5.3 software. The treatment effect will be estimated as weighted mean difference or standardized mean difference and 95% confidence intervals (CIs) for continuous data, and risk ratio and 95% CIs for dichotomous data. Statistical heterogeneity will be examined by *I*^2^ test. The values of *I*^2^ ≤50% show fair homogeneity and a fixed-effects model will be applied. On the other hand, the values of *I*^2^ > 50% mean obvious heterogeneity, and a random-effects model will be practiced. If homogeneity is identified, we will perform a meta-analysis when sufficient data are extracted. If significant heterogeneity is examined, we will carry out a subgroup analysis to find out sources of heterogeneity.

#### 2.8.2 Subgroup analysis

Subgroup analysis will be suggested to explore possible reasons for the substantial heterogeneity in accordance with different types of treatments, controls, and outcome measurements.

#### 2.8.3 Sensitivity analysis

Sensitivity analysis will be performed to test the robustness of study results by eliminating low quality studies.

#### 2.8.4 Reporting bias

We will carry out a funnel plot and Egger's regression test to check reporting bias when at least 10 trials are included.^[24]^

### 2.9 Grading the quality of evidence

The evidence level for main outcomes will be appraised by two independent researchers using Grading of Recommendations Assessment, Development, and Evaluation.^[25]^ Any divisions will be solved by a third researcher through discussion.

## 3 Discussion

Previous studies have reported the effect of grelin on TRXE in CHFT. However, to the best of our knowledge, no study has systematically performed this topic, and thus no high quality evidence-based medicine has been provided to determine whether grelin is effective on TRXE in CHFT. Thus, this systematic review will utilize stringent eligibility criteria to evaluate the effect of grelin on TRXE in CHFT. The results of this study will provide clear evidence to judge whether grelin is effective on TRXE in CHFT, and will be beneficial to clinical practice and further studies.

## Author contributions

**Conceptualization:** Zhe Chen, Ru-bing Zhang, Xin Li, Jian-bo Xu.

**Data curation:** Zhe Chen, Yao Feng.

**Formal analysis:** Zhe Chen, Yao Feng, Ru-bing Zhang, Xin Li.

**Investigation:** Jian-bo Xu.

**Methodology:** Zhe Chen, Yao Feng, Ru-bing Zhang.

**Project administration:** Jian-bo Xu.

**Resources:** Zhe Chen, Yao Feng, Ru-bing Zhang, Xin Li.

**Software:** Zhe Chen, Yao Feng, Ru-bing Zhang, Xin Li.

**Supervision:** Jian-bo Xu.

**Validation:** Zhe Chen, Yao Feng, Xin Li, Jian-bo Xu.

**Visualization:** Zhe Chen, Ru-bing Zhang, Xin Li, Jian-bo Xu.

**Writing – original draft:** Zhe Chen, Yao Feng, Ru-bing Zhang, Jian-bo Xu.

**Writing – review & editing:** Zhe Chen, Xin Li, Jian-bo Xu.
